# Unveiling photoinduced electron transfer in cobalt(iii)-R-pyridine complexes anchored to anatase nanocrystals: photoluminescence and magnetic studies†

**DOI:** 10.1039/d4ra02648a

**Published:** 2024-05-28

**Authors:** Ganeshraja Ayyakannu Sundaram, Krishnamoorthy Anbalagan, Mohammad Ahmad Wadaan, Jagan Rajamoni, Vaithinathan Karthikeyan

**Affiliations:** a Department of Research Analytics, Saveetha Dental College and Hospitals, Saveetha Institute of Medical and Technical Sciences Poonamallee High Road Chennai Tamil Nadu 600 077 India ganeshrajaas.sdc@saveetha.com asgchem84@gmail.com; b Department of Chemistry, Pondicherry University Kalapet Pondicherry 605 014 India kanuniv@gmail.com; c Department of Zoology, College of Science, King Saud University P. O. Box. 2455 Riyadh 11451 Saudi Arabia; d Department of Chemistry and Biochemistry, University of Missouri St. Louis MO 63121 USA; e School of Science and Technology, Hong Kong Metropolitan University Ho Man Tin Hong Kong vkarthik@hkmu.edu.hk

## Abstract

In this study, we synthesized mixed ligand complexes of the *cis*-[Co(tn)_2_(Rpy)Br]Br_2_ type using a novel mechanochemical approach. Characterization involved spectral measurements and single crystal X-ray diffraction analysis, confirming the structure of the *cis*-[Co(tn)_2_(4-Mepy)Br]Br_2_ complex. The single crystal refinement data revealed a monoclinic crystal system with a distorted octahedral geometry. The choice of the sixth ligand influenced the emission and magnetic properties, showing a ferromagnetic character in the Co(iii)-complex environment. We investigated efficient electron transfer to the cobalt(iii) center using TiO_2_ nanoparticles under UV-light irradiation. The adsorption characteristics of *cis*-[Co(tn)_2_(Rpy)Br]Br_2_ in aqueous 2-propanol varied, leading to surface compound formation. Under UV irradiation, the anatase surface exhibited remarkable adsorption capabilities, facilitating efficient electron transfer to the Co(iii) center and resulting in a high photoefficiency for Co(ii) formation. Our study has put forward a model for interfacial electron transfer (IET), taking into account the overlap between the TiO_2_ conduction band and the acceptor level of the Co center, as well as the electronic coupling between the donor level of the Ti center and the acceptor level of the Co center. This model sheds light on the accumulation of electrons for reducing the adhered complex ion. The IET process was corroborated by the conversion of 2-propanol into acetone, as verified by ^1^H NMR technique. Overall, our findings provide novel insights into the role of the Rpy moiety in modifying the structure of the TiO_2_-cobalt(iii)-Rpy compound and propose a mechanism for IET reactions, thus advancing the field.

## Abstract 2

This article demonstrates the synthesis of mixed ligand complexes of *cis*-[Co(tn)_2_(Rpy)Br]Br_2_*via* a mechanochemical approach. Their ferromagnetic properties and efficient electron transfer to Co(iii) using TiO_2_ under UV light are confirmed.

## Graphical abstract

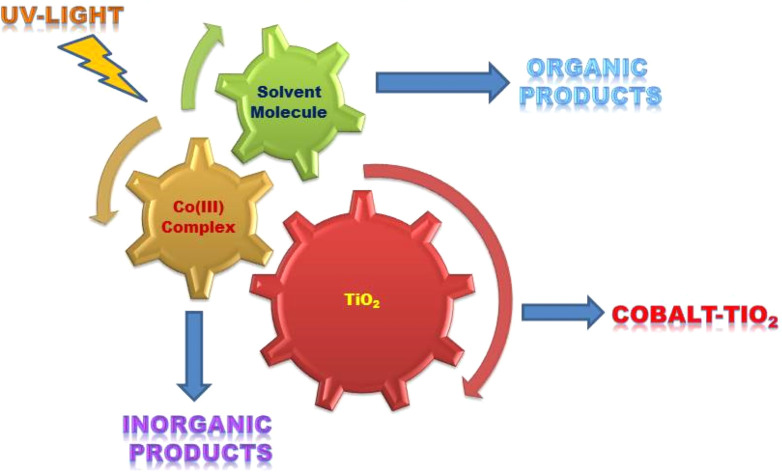

## Introduction

Understanding the intricate photophysics of transition metal complexes tethered to semiconductor surfaces is crucial for designing artificial systems for solar energy conversion.^1^ Cobalt(iii) compounds have emerged as promising candidates due to their ability to foster long-lived charge-separated states and exhibit rich photochemical properties.^2,3^ However, the electronic excitations and photoconversion mechanisms of these complexes are only partially understood.^4,5^ Beyond their application in photovoltaic cells for converting solar energy into electricity, Ru(ii)-polypyridine complexes anchored to TiO_2_ surfaces *via* phosphonate linkers exhibit remarkable resistance to humidity and can emulate photosynthetic charge transfer events.^6,7^ Consequently, unraveling the intricacies of electron transfer mechanisms stands to impact the development of multicomponent molecular assemblies for photocatalysis and artificial photosynthesis. Previous theoretical endeavors have primarily focused on the comparative analysis of binding energies and vertical excitations, particularly when covalently attaching [Ru(tpy)_2_]^2+^ to TiO_2_ using carboxylates or phosphonate linkers.^8,9^ Similar investigations have extended to the attachment of dye-sensitized TiO_2_ nanoparticles.^10,11^

Estimates of electron injection rates within the time-independent framework have been derived from the analysis of donor and acceptor orbital overlap in both adsorbate and host-nanoparticle. Meanwhile, explicit quantum dynamics simulations have probed the time scales and mechanisms of interfacial electron transfer (IET), building upon earlier studies of electron injection in sensitized TiO_2_ semiconductors.^12^ The reported results herein offer fundamental insights into the electronic and structural properties of molecular assemblies, crucial for achieving efficient IET in catalyst-chromophore/TiO_2_ interfaces.^13,14^

Recent research endeavors outlined in this discussion have been spurred by the pursuit of advancing photovoltaic cells built upon sensitized nanocrystalline TiO_2_.^15–18^ Notably, investigations in this domain have revealed that while the fundamental photophysical properties of molecular components persist upon immobilization on a semiconducting surface, the interaction with said surface can significantly modulate the rates of individual photophysical processes.^19–21^ A compelling illustration of this phenomenon is observed in ruthenium polypyridyl complexes, which, when bound to TiO_2_, undergo a remarkable transformation from intrinsic photolability in solution to pronounced photostability.^22,23^ This transformation is ascribed to the exceptionally rapid sub-picosecond charge injection from the excited state of the surface-bound compound into the conduction band of the semiconductor.^24^ Furthermore, the subsequent back electron transfer process, involving conduction band electrons and the oxidized form of the sensitizer, occurs at a significantly slower rate than the forward electron transfer reaction.^25^ Consequently, this pronounced disparity in rates facilitates effective charge separation, rendering the TiO_2_ surface instrumental in achieving long-lived charge separation—a pivotal factor driving investigations in this field.^26^

In this study, we utilized a novel mechanochemical approach to synthesize *cis*-[Co(tn)_2_(Rpy)Br]Br_2_ mixed ligand complexes. Advanced instrumental analyses were employed to explore the geometry and the impact of aryl ligands on the emission and magnetic properties within the coordination sphere. The primary focus lies on investigating the luminescent and magnetic characteristics of these complexes, which also provide access to stable cobalt(iii) monomers. Specifically, we aim to comprehensively understand the redox activity of excited nanocrystalline TiO_2_ and structurally modified cobalt(iii)-aryl amine complexes, particularly focusing on the influence of ligand electron donor strength on the cobalt(iii) complex's M−H loop. Our objective is to unravel the intricate interplay between photoinduced electron transfer phenomena and cobalt(iii)-R-pyridine complexes immobilized onto anatase nanocrystals. This comprehensive study includes detailed synthesis methodology, characterization techniques, and analysis of photoluminescence and magnetic properties. By exploring complex interfacial dynamics, our research sheds light on unique electron transfer processes within this system, offering valuable insights to photochemical and materials science. Ultimately, the custom-designed metal complexes developed here hold promise for boosting photocatalytic activity and photon harvesting by facilitating efficient interfacial electron transfer processes. Notably, the validity of the IET process was confirmed through the conversion of 2-propanol into acetone, as validated by ^1^H NMR technique.

## Experimental section

### Materials and instrumental methods

Reagent-grade cobaltous chloride, 1,3-propanediamine (tn), *R*-pyridine (analytical reagent grade), KBr (spectral grade), nanocrystalline titanium dioxide (with a BET surface area of 150–200 m^2^ g^−1^, particle size of approximately 50 nm, and micropore volume of ∼0.027 cm^3^ g^−1^), and DMSO-d_6_ (spectral grade) were procured from Sigma-Aldrich and utilized without further purification. Triply distilled water was obtained using an all-glass apparatus over alkaline potassium permanganate. Elemental analyses were performed using an Elementar Vario EL III-Germany instrument.

FT-IR spectra of the complexes were acquired using a Thermo Nicollet-6700 FT-IR instrument in the range of 4000–400 cm^−1^ (KBr pellet technique). Electronic absorption spectral studies were conducted on a double-beam spectrophotometer (Shimadzu 2450, Japan) with an integrating sphere attachment (ISR-2200).

Single crystals of the complex were examined using an Oxford Diffraction Xcalibur diffractometer with an Eos (Nova) detector, utilizing ω and φ scan modes. Diffraction measurements were carried out at 293(2) K using graphite monochromated Mo-Kα radiation (*λ* = 0.71073 Å). Structure solution and refinement were accomplished by direct methods and full-matrix least squares on F2, respectively, utilizing the 32 bit Olex 2-1.1 version program. Other computer programs involved in data collection, cell refinement, data reduction, and absorption correction included CrysAlis PRO (Oxford Diffraction, 2009).

Steady-state fluorescence emission was recorded on a Spex FluoroLog-3 spectrofluorometer (Jobin-Yvon Inc.) equipped with a 450 W Xenon lamp. Time-resolved fluorescence decay measurements were conducted using a Nano-LED (*λ*_ex_ = 295 nm) source for excitation (repetition rate 10 kHz). Photon collection utilized a TBX-4-X single-photon-counting detector, and lifetimes were determined by fitting the data to exponential decay models. The fitting analysis was performed using DAS6 v6.2-Horiba Jobin Yvon software, and magnetic measurements were conducted using a vibrating sample magnetometer (VSM) in powder form on Lakeshore-7404. The VSM technique allowed the observation of hysteresis curves for the all prepared complexes in a DC magnetic field.

^1^H NMR spectra of the acetone formation were obtained on a Bruker instrument, model Avance-II, operating at 400 MHz (∼9.4 Tesla) for Fourier-transform nuclear magnetic resonance. The complex was dissolved in DMSO-d_6_ with added TMS as an internal standard, and the solution was placed in an NMR tube for ^1^H NMR. For ^1^H NMR spectra, measurements comprised 256 scans with a 3.52 s repetition time.

### Synthesis of *cis*-[Co(tn)_2_(Rpy)Br]Br_2_

**Fig. 1:**
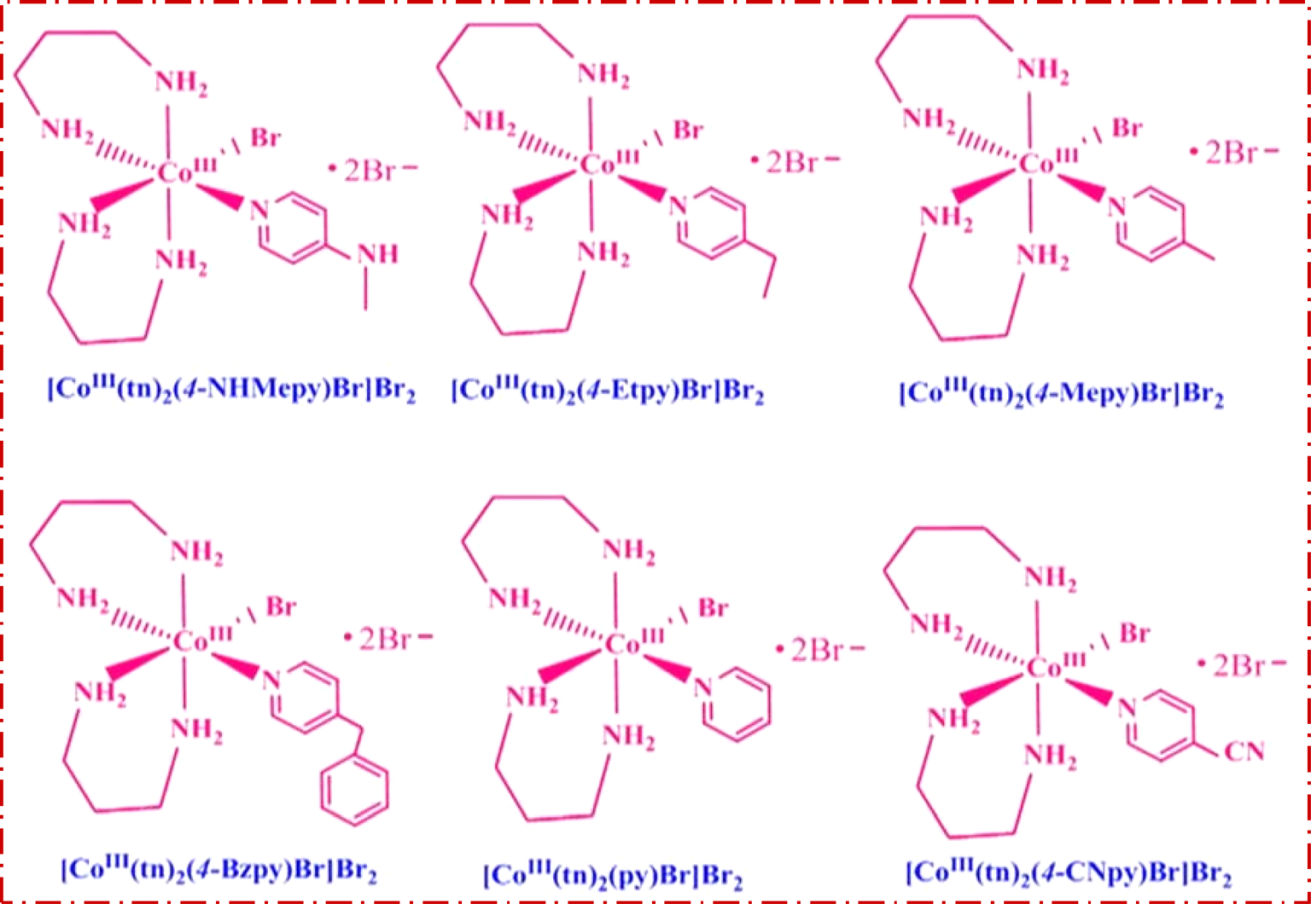
Structure of the *cis*-[Co(tn)_2_(Rpy)Br]Br_2_ complexes.

*Trans*-[Co(tn)_2_Br_2_]Br, precursor complex was synthesized following the established procedure in the literature.^27–29^ The *cis*-[Co(tn)_2_(Rpy)Br]Br_2_ complexes were synthesized utilizing an simple mechanochemical approach from the precursor complex. To this end, one gram of *trans*-[Co(tn)_2_Br_2_]Br was transformed into a paste using distilled water and subsequently ground with an equimolar amount of *R*-pyridyl (where R = 4-CN, H, 4-Bz, 4-Me, 4-Et, and 4-MeNH) for 3–5 hours. The resulting mixture was left undisturbed overnight in a dark environment, and the solid product obtained underwent washing with ethanol before being recrystallized using distilled water. The structural details of the *cis*-[Co(tn)_2_(Rpy)Br]Br_2_ complexes are illustrated in Fig. 1.

### *cis*-[Co(tn)_2_(4-MeNHpy)Br]Br_2_ (1)

Recrystallized from water to give dark pink colored powder, yield 0.813 g, 81%. Anal. calc. for C_12_H_28_CoN_6_Br_3_: C 25.97, H 5.09, N 15.14, Br 43.19%. Found: C 25.23, H 4.93, N 14.33, and Br 43.11%. FTIR (KBr disc) cm^−1^, 3504.06(b): O–H stretching vibrations of coordinated or adsorbed water molecules, 3207.16(w): stretching vibration of NH groups, 3070.63(w): stretching vibration of aromatic C–H bonds., 1613.25(s): the stretching vibration of the C=N bond in the pyridine ring, 1416.68(s): the bending vibration of N–H bonds or C=C vibrations, 1305.65(w): stretching vibration of C–N bonds, 1229.98(m): C–N stretching vibrations, 1156.79(w): C–H bending vibrations, 1100.04(w): C–H bending vibrations, 1072.89(m): to stretching vibrations of C–N bonds or C–H bending vibrations, 1012.03(m): stretching vibrations of C–N bonds, 928.97(w): C–H bending vibrations, 826.98(s): bending vibrations of C–H bonds, 757.89(w), 697.04(w), 651.80(w), 594.23(w) and 522.68(w): bending vibrations of C–H bonds or metal–ligand vibrations. UV-vis (water 1.80 × 10^−3^ M) *λ*_max_, nm (*ε*_max_ M^−1^ cm^−1^), 359.9 (1.23 × 10^2^), 518.3 (90.47).

### *cis*-[Co(tn)_2_(4-Etpy)Br]Br_2_ (2)

Recrystallized from water to give dark pink colored powder, yield 0.724 g, 72%. Anal. calc. for C_13_H_29_CoN_5_Br_3_: C 28.18, H 5.28, N 12.64, Br 43.26%. Found: C 27.55, H 4.83, N 12.41, and Br 42.21%. FTIR (KBr disc) cm^−1^, 3222.63(b): O–H stretching vibrations of coordinated water molecules, 1680.51(w): the stretching vibration of the C=N bond, 1433.07(s): the bending vibration of N–H bonds or C=C vibrations, 1144.43(s): stretching vibrations of C–N bonds or C–H bending vibrations, 1088.11(s): stretching vibrations of C–N bonds, 980.16(w): stretching vibrations of C–N bonds, 625.31(m), 552.55(w) and 457.53(w): bending vibrations of C–H bonds or metal–ligand vibrations. UV-vis (water 1.84 × 10^−3^ M for UV region 1.84 × 10^−4^ M for visible region) *λ*_max_, nm (*ε*_max_ M^−1^ cm^−1^), 265.06 (4.34 × 10^3^), 358.64 (1.23 × 10^3^), 516.30 (8.85 × 10^2^).

### *cis*-[Co(tn)_2_(4-Mepy)Br]Br_2_ (3)

Recrystallized from water to give dark pink colored powder, yield 0.867 g, 87%. Anal. calc. for C_12_H_27_CoN_5_Br_3_: C 26.69, H 5.04, N 12.97, Br 44.39%. Found: C 26.35, H 4.83, N 12.33, and Br 44.11%. FTIR (KBr disc) cm^−1^, 3437(b) and 3350.90(b): O–H stretching vibrations of coordinated water molecules, 1616.61(s): the stretching vibration of the C=N bond in the pyridine ring, 1558.71(w): stretching vibrations of C–N bonds or C–H bending vibrations, 1503.62(m), 1443.28(m), 1424.97(m) and 1382.80(w): bending vibrations of N–H bonds or C=C vibrations, 1230.69(m) and 1213.30(m): stretching vibrations of C–N bonds or C–H bending vibrations, 1072.04(w): stretching vibrations of C–N bonds or C–H bending vibrations, 1020.47(m) and 987.86(w): stretching vibrations of C–N bonds or C–H bending vibrations, 813.15(s): bending vibrations of C–H bonds, 722.99(m), 597.51(w) and 539.55(w): bending vibrations of C–H bonds, 493.71(s): bending vibrations of C–H bonds or metal–ligand vibrations. UV-vis (water 1.89 × 10^−3^ M for UV region 1.89 × 10^−4^ M for visible region) *λ*_max_, nm (*ε*_max_ M^−1^ cm^−1^), 256.06 (6.86 × 10^4^), 357.92 (1.22 × 10^3^), 512.83 (8.45 × 10^2^).

### *cis*-[Co(tn)_2_(4-Bzpy)Br]Br_2_ (4)

Recrystallized from water to give dark pink colored powder, yield 0.793 g, 79%. Anal. calc. for C_18_H_32_CoN_5_Br_3_: C 35.03, H 5.23, N 11.35, Br 38.84%. Found: C 34.84, H 4.82, N 11.12, and Br 38.19%. FTIR (KBr disc) cm^−1^, 3732.52(w) and 3443.47(b): stretching vibrations of O–H bonds, likely from coordinated water molecules, 3212.84(w), 3119.87(w), 3060.38(w) and 3030.58(w): stretching vibrations of N–H bonds, 1662.80(w) and 1611.95(s): the stretching vibration of the C=C and C=N bonds in the benzene and pyridine rings, respectively, 1556.84(m), 1496.24(s), 1452.26(m) and 1423.59(s): bending vibrations of N–H bonds or C=C vibrations, 1282.93(w) and 1223.04(s): stretching vibrations of C–N bonds, 1115.93(w), 1070.16(m), 1037.82(w), 1014.47(m), 925.34(w), 856.26(w) and 835.56(w): stretching vibrations of C–N bonds or C–H bending vibrations, 795.36(s), 743.79(s), 700.85(s), 670.81(w), 644.32(w) and 617.02(s): bending vibrations of C–H bonds, 559.30(m), 486.93(s) and 459.55(w): C–H bonds or metal–ligand vibrations. UV-vis (water, 1.77 × 10^−3^ M for UV region 1.77 × 10^−4^ M for visible region) *λ*_max_, nm (*ε*_max_ M^−1^ cm^−1^), 257.21 (3.76 × 10^4^), 350.26 (1.57 × 10^3^), 516.30 (7.56 × 10^2^).

### *cis*-[Co(tn)_2_(py)Br]Br_2_ (5)

Recrystallized from water to give dark pink colored powder, yield 0.871 g, 87%. Anal. calc. for C_11_H_25_CoN_5_Br_3_: C 25.12, H 4.79, N 13.32, Br 45.57%. Found: C 24.55, H 4.33, N 13.23, and Br 44.91%. FTIR (KBr disc) cm^−1^, 3219.35(w), 3164.02(w), 3081.60(w) and 2925.59(w): stretching vibrations of N–H bonds, 1603.37(w) and 1562.07(w): stretching vibrations of the C=N bond in the pyridine ring, 1435.35(s): bending vibrations of N–H bonds or C=C vibrations, 1402.79(m), 1321.34(w), 1289.22(w) and 1250.70(w): bending vibrations of N–H bonds or C–H bending vibrations, 1143.89(s), 1095.21(s), 981.21(w), 926.11(w) and 886.70(w): stretching vibrations of C–N bonds or C–H bending vibrations, 738.33(w), 625.21(m), 553.07(w), 520.69(w) and 450.04(w): bending vibrations of C–H bonds or metal–ligand vibrations. UV-vis (water 3.17 × 10^−3^ for UV region 3.17 × 10^−4^ M for visible region) *λ*_max_, nm (*ε*_max_ M^−1^ cm^−1^), 255.39 (1.02 × 10^4^), 351.57 (8.42 × 10^2^), 514.86 (4.15 × 10^2^).

### *cis*-[Co(tn)_2_(4-CNpy)Br]Br_2_ (6)

Recrystallized from water to give dark pink colored powder, yield 0.820 g, 82%. Anal. calc. for C_12_H_25_CoN_6_Br_3_: C 26.11, H 4.57, N 15.22, Br 43.42%. Found: C 25.55, H 4.13, N 15.03, and Br 42.82%. FTIR (KBr disc) cm^−1^, 3473.63(b): O–H stretching vibrations of coordinated water molecules, 3199.76(w), 3070.63(w) and 2941.51(w): stretching vibrations of N–H bonds, 1944.69(w): stretching vibrations of the C≡N bond in the cyano group, 1716.87(s): stretching vibrations of the C=N bond in a carbonyl group, 1670.81(m), 1602.55(s), 1549.10(m), 1465.21(m), 1423.26(s), 1373.91(w) and 1321.28(w): bending vibrations of N–H bonds or C–H bending vibrations, 1229.98(m), 1146.09(w), 1069.61(w), 1016.97(m), 933.07(w), 826.98(m), 757.89(w), 700.32(w) and 644.40(w): stretching vibrations of C–N bonds or C–H bending vibrations, 537.48(m): bending vibrations of C–H bonds or metal–ligand vibrations. UV-vis (water 1.81 × 10^−3^ M) *λ*_max_, nm (*ε*_max_ M^−1^ cm^−1^), 357.9 (1.26 × 102), 512.1 (89.44).

Note: complex numbers from (1) to (6) have been assigned to each molecular formula provided in the above section. Hereafter, these complex numbers refer to the respective complexes.

### Photocatalytic reduction of Co(iii)-R-pyridine complexes

The IET process was investigated through the photoreduction of the *cis*-[Co(tn)_2_(Rpy)Br]Br_2_ complex employing TiO_2_ nanocrystals in water/aqueous 2-propanol solutions under 254 nm light irradiation. The photoefficiency of Co^2+^ ion formation from the above system was estimated using Kitson's method.^30^ Photoreduction experiments were conducted in a reactor vessel utilizing a 254 nm low-pressure mercury vapor lamp, housed in a Lab Guard fume hood and covered with a black polythene sheet to eliminate extraneous light. The photoreactor, a double-walled quartz vessel, contained a photolyte mixture of 100 mg of TiO_2_ nanocrystals in 80 mL of aqueous 2-proponal solution of *cis*-[Co(tn)_2_(Rpy)Br]Br_2_ (1.98 × 10^−3^ M), and 10 mL of 1 M NaNO_3_ (for stabilize pH) in the inner jacket, with cool water in the outer jacket. Before irradiation, the catalyst suspension was achieved through ultrasonic treatment and continuous magnetic stirring in the dark to attain equilibrium in cobalt(iii) complex ion adsorption/desorption on the catalyst. The photolyte suspension was exposed to 254 nm light for specific irradiation periods (2–16 min). Subsequently, 4 mL aliquots were sampled, centrifuged to remove solid particles, and then subjected to spectral analysis. To minimize experimental error, each experiment was replicated at least three times for the same sample, and the mean value was calculated. The photoefficiency of Co^2+^ formation, expressed as a percentage (PE %), was determined using the formula: PE (%) = [(*A*_*t*_ − *A*_i_)/*A*_*t*_] × 100, where *A*_i_ and *A*_*t*_ are the absorbances of the photolysed solutions initially and at a defined time interval ‘*t*’, respectively.

## Results and discussion

### Characterization of cobalt(iii)-R-pyridyl complexes

The cobalt(iii)-R-pyridine complexes, *cis*-[Co(tn)_2_(Rpy)Br]Br_2_, has been successfully synthesized, and its structural characteristics were comprehensively elucidated through a multi-faceted analytical approach. The determination of the complex's structure employed techniques such as FTIR, UV-vis spectroscopy, luminescence studies, and, notably, single crystal X-ray crystallography. The application of FTIR and UV-vis spectroscopy provided valuable insights into the molecular composition and electronic properties of the synthesized complex. Luminescence studies further probed the optical properties, adding a dimension to our understanding of the complex's behavior in excited states. However, the most definitive evidence supporting the cis structure of the aforementioned complexes was derived from the meticulous examination of their crystal structure using single crystal X-ray crystallography. This technique, known for its unparalleled precision in revealing molecular arrangements, unambiguously confirmed the *cis* configuration of the cobalt(iii)-pyridine complex. The synthesis and structural elucidation of the *cis*-[Co(tn)_2_(Rpy)Br]Br_2_ complex were conducted through a synergistic application of diverse analytical methods, with single crystal X-ray crystallography serving as the definitive tool in confirming the cis configuration of the complex.

**Fig. 2:**
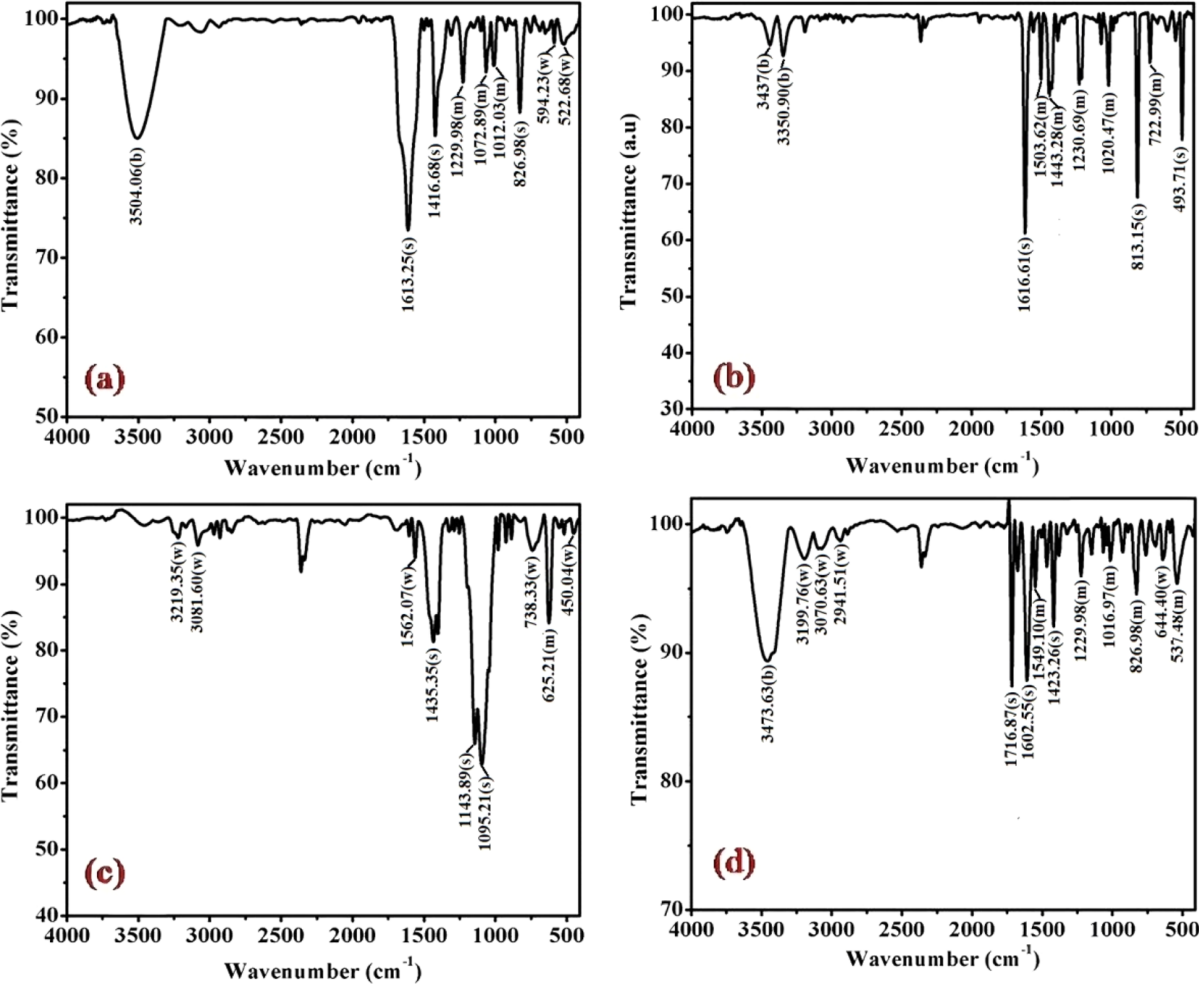
Displays the FTIR spectra of complexes (a) (1), (b) (3), (c) (5), and (d) (6), recorded in KBr at 298 K.

The FT-IR spectra of complexes (1), (3), (5) and (6) are depicted in Fig. 2 and for remaining complexes (2) and (4) in Fig. S1.† Analysis of the IR data reveals distinctive peaks in the range of 3500 to 3400 cm^−1^, attributable to the antisymmetric and symmetric *ν*(O–H) stretching modes of lattice-bound water.^31^ The existence of stretching absorption bands within the range of 1650–1620 cm^−1^ affirms bending modes associated with *ν*(O–H), signifying the potential presence of the hydrated form of the molecule through hydrogen bonding in some of the complexes.^26,32^ However, it's important to note that this characteristic is not illustrated in the single crystal studies of the representative complex (4) discussed in the subsequent sections.

Significant shifts in the antisymmetric *ν*(N–H) stretching modes at ∼3318–3005 cm^−1^ (metal-amine at ∼3300 cm^−1^) and deformation mode at ∼1632–1497 cm^−1^ (metal-amine ∼1600 cm^−1^) provide clear evidence of cobalt-amine coordination. Typically, [Co(N–N)_2_LX]^2+^ type molecules exhibit vibrations at 910–800 cm^−1^ in the –CH_2_ rocking region, with cis complexes manifesting more bands in this region than trans complexes.^31,32^ In this study, bands at ∼812, ∼868, and ∼910 cm^−1^ confirm the –CH_2_ rocking region, affirming the coordination of 1,3-propanediamine molecules to the metal ion. The band at 1617 cm^−1^ is assigned to the heterocyclic ring vibration, indicating coordination of the nitrogen atom of the 4-substituted pyridine ligand with the metal ion. Newly observed strong bands in the 492 cm^−1^ range may be attributed to M−N stretching. Bands in the 1320 cm^−1^ region, corresponding to NH_2_ deformation, further confirm the structural similarity of the Co(iii) complexes to the earlier proposed [Co(en)_2_(RNH_2_)Cl]^2+^ ion.^31^ Distinctive bands at 1458 cm^−1^ and 1578–1590 cm^−1^ are observed, corresponding to C=C and C=N vibrations of the pyridine ring, respectively. Additionally, bands at ∼589 cm^−1^ and ∼590 cm^−1^ corresponding to Co–N(tn) and Co–N of NH_2_(tn) bending, respectively, are exhibited around 1650 cm^−1^.^32^ The FTIR spectral studies indicate that the remaining spectra align with identical peak patterns, as depicted in Fig. S1.† Confirmation from these studies suggests the presence of tn and R-py moieties within the coordination sphere, with a high likelihood of adopting a cis configuration, barring exceptions.

**Fig. 3:**
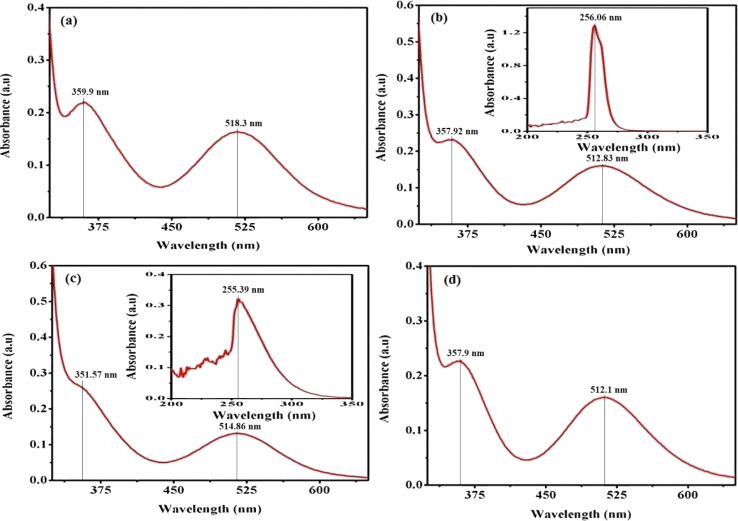
Presents the UV-vis electronic absorption spectra of complexes (a) (1), (b) (3), (c) (5), and (d) (6), recorded in water (300 μM) at 298 K. The insert focuses on the 200–350 nm range.

In practice, the identification of trans structures is often associated with a weak band in the range of approximately 625–700 nm, whereas the corresponding *cis*-CoL_4_*X*_2_ complexes typically exhibit absorption around 550 nm.^32^ The [Co(tn)_2_(Rpy)Br]Br_2_ complex manifests two distinct bands at 359 nm and approximately 516 nm, as illustrated in Fig. 3. Consistent with this pattern, the spectra of the remaining complexes exhibit analogous peaks, as depicted in Fig. S2.† The absorption bands at around 360 nm in these complexes are attributed to the ligand-to-metal charge transfer (LMCT) band, while those at approximately 515 nm correspond to the first absorption band, specifically the ^1^A_1_g → ^1^T_1_g transition. The observed positions and intensities strongly indicate a cis configuration rather than a trans structure for these complexes.^33^ Additionally, the electronic spectrum of these complexes bears resemblance to that of the *cis*-β-[Co(trien)Cl]^2+^ ion, suggesting a potential *cis*-β arrangement of ligands around the Co(iii) ion.^34^

Furthermore, the positions of these bands vary across the series of complexes. In cobalt(iii) complexes, regular spin-allowed transitions are typically ^1^A_1_ → ^1^T_1_ and ^1^A_1_ → ^1^T_2_, which split into components for complexes with lower symmetry, such as [Co(tn)_2_(Rpy)Br]^2+^, resulting in ^1^A_1_ → ^1^A_2_ + ^1^E_a_ and ^1^A_1_ → ^1^B_2_ + ^1^E_b_ transitions. Notably, among these transitions, only the ^1^A_1_ → ^1^B_2_ is magnetically forbidden.^35^ The higher energy visible band is centered at 360 nm, while the low-energy band is expected around ∼515 nm, though the latter is obscured under the conditions of the present experimental setup.

The complex ion, [Co(tn)_2_(Rpy)Br]Br_2_, exhibits a prominent band in the UV region. This phenomenon is interpreted through the lens of the linear combination of atomic orbitals - molecular orbital (LCAO-MO) theory, suggesting that these bands result from charge transfer events involving the p-orbitals of the halide ion and predominantly the dz^2^ orbital of the cobalt(iii) ion. The π → π* transition attributed to the Rpy ligand occurs at a shorter wavelength, approximately 244 nm, as illustrated in the inset of Fig. 3(b) and (c). It's worth noting that at this concentration, the detection of the high-energy charge transfer band is either obscured in some cases or not observed.^36^

In pyridine systems, optical transitions from the highest occupied molecular orbital (HOMO) to the lowest unoccupied molecular orbital (LUMO), π → π*, typically occur at 244 nm. However, the presence of acceptor/donor groups attached to the ring can alter the energies of LUMO/HOMO levels. The intensities of absorption due to d–d bands in [Co(tn)_2_(Rpy)Br]^2+^ parallel the magnitude of *ε* for [Co(en)(NH_3_)_3_CN]^2+^ ion.^37^ Notably, it has been reported that the molar extinction coefficient of the d–d band increases approximately tenfold when *X* is substituted by Cl → Br → I.^32,38^ Crucially, a weak band around 515 nm serves as a characteristic feature of *cis* structures.^39^ Moreover, cobalt(iii) complexes with lower symmetry and aromatic amines are inclined to exist in the *cis*-form, making the cis configuration more probable in this context.

Upon comparing the various peaks observed in complexes (1) through (6), it becomes apparent that there is no discernible consistency in changes from complex (1) to complex (2). This lack of uniformity suggests that the sixth ligand of Rpy does not instigate systematic alterations in the optical characteristics of our synthesized complexes. However, a more in-depth examination reveals an intriguing trend: there is an approximate shortening of absorption wavelengths for both charge transfer bands and d–d bands from complex (1) with R = 4-MeNH ligand to complex (6) with R = 4-CN ligand in the sixth ligand of RPy within our prepared complexes. This trend is illustrated in Fig. 3.

This observed shift suggests that the presence of a releasing group in RPy leads to longer absorption wavelengths compared to a withdrawing group. Despite this influence, it's important to note that the sixth ligand's impact on the optical behavior of our complexes is modest and lacks a consistent pattern across the series. This indicates that while the ligand does contribute to variations in optical properties, there are likely other factors at play influencing the overall optical behavior. Further investigation into these factors could provide deeper insights into the underlying electronic structure governing the optical properties of these complexes.

### Single crystal X-ray diffraction analysis

The single crystal refinement data for complex (3) reveals a symmetric structure with a twofold rotation axis, belonging to the monoclinic crystal system. The refined unit cell parameters are as follows: *a* = 9.058(2) Å, *b* = 16.135(4) Å, *c* = 13.128(3) Å, *α* = *γ* = 90°, *β* = 92.61(2)°, and a volume of 1916.68 Å^3^. The crystal structure adopts the space group *P*2_1_/*n*, and the unit cell accommodates four formula units (*z* = 4). Additionally, further insights into the crystallographic details and intermolecular interactions within the complex are provided in the subsequent analysis.

**Fig. 4:**
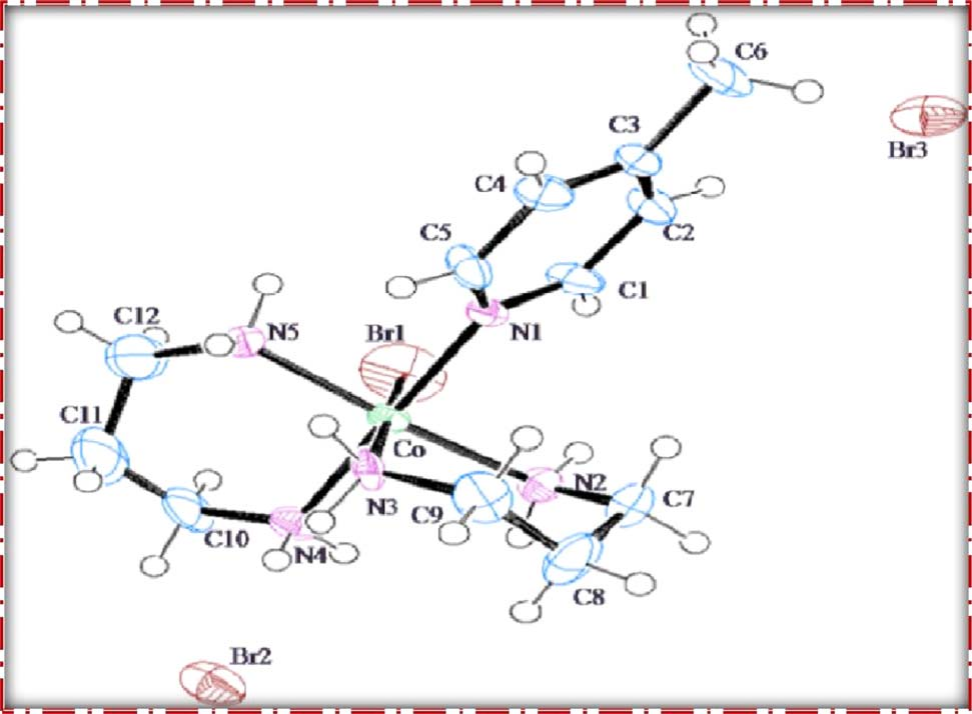
ORTEP diagram of [Co(tn)_2_(4-Mepy)Br]Br_2_ (3) complex.

**Fig. 5:**
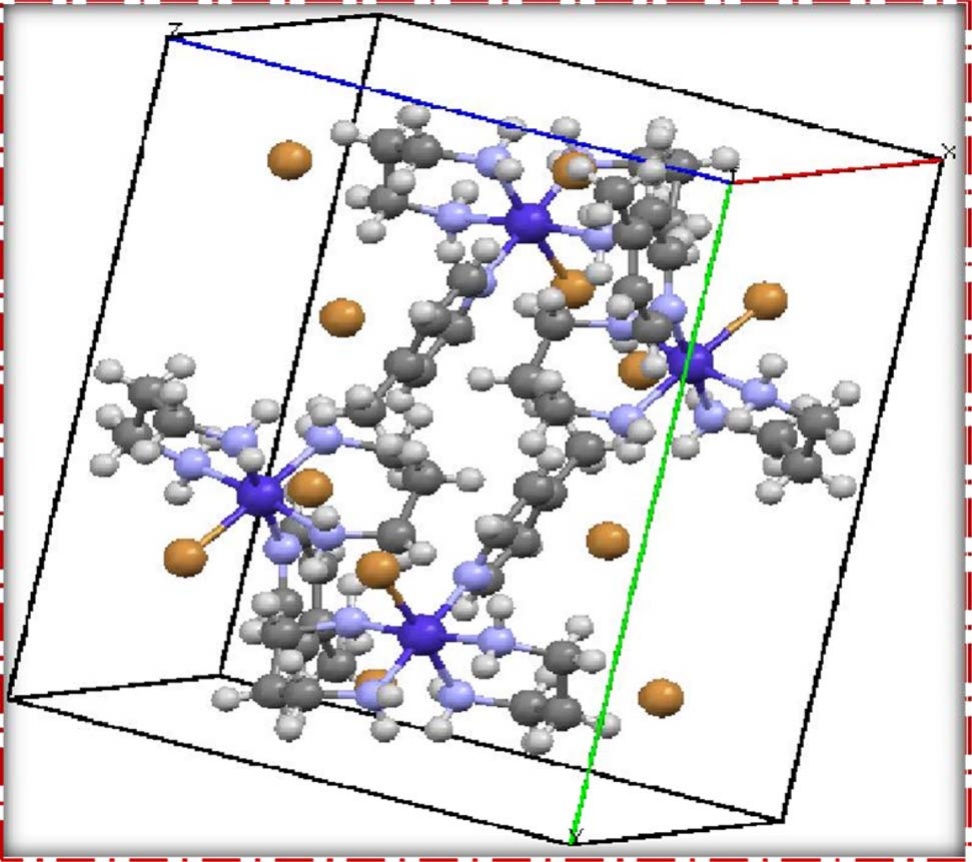
Packing diagram of [Co(tn)_2_(4-Mepy)Br]Br_2_ (3) complex.

The crystal structure of the obtained cobalt(iii) complex was elucidated through X-ray diffraction, revealing an octahedral coordination environment for the complex (3) complex. The single-crystal X-ray diffraction refinement affirmed that the molecule is classified under the monoclinic crystal system, featuring a distorted octahedral geometry, as illustrated in Fig. 4. Detailed insights into the molecular arrangement, bond angles, and interatomic distances were gleaned from this comprehensive structural analysis. Notably, the packing diagram derived from the X-ray diffraction data, as depicted in Fig. 5, highlights the absence of significant intermolecular interactions. This information is pivotal in understanding the spatial arrangement of individual molecules within the crystal lattice.

Moreover, the single crystal X-ray diffraction studies provided crucial validation for the structural integrity of our prepared representative complex. It is anticipated that a similar structural motif is maintained across the remaining complexes synthesized in this study, reinforcing the reliability and consistency of the synthetic methodology employed. The wealth of information obtained from these crystallographic analyses lays a robust foundation for the subsequent interpretation of the complex's properties and behaviors.

### Fluorescence study of [Co(tn)_2_(Rpy)Br]Br_2_

**Fig. 6:**
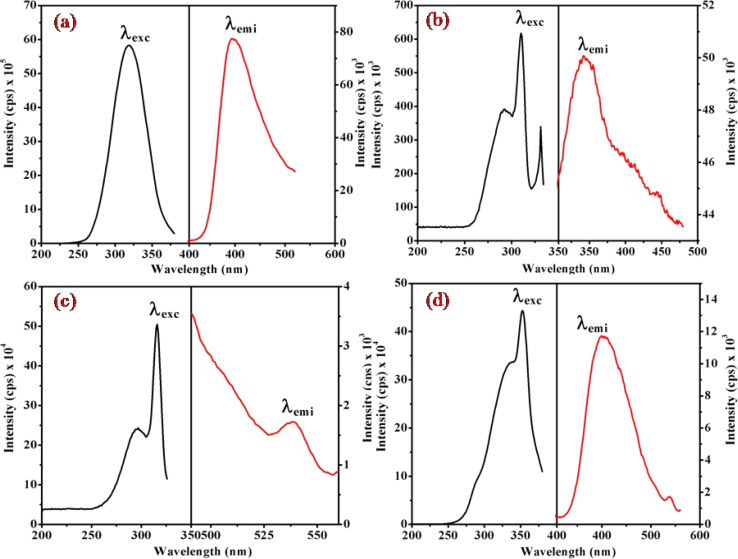
Presents the steady-state excitation and emission spectra of complexes (a) (1), (b) (3), (c) (5), and (d) (6), recorded at *λ*_max_ = 256 nm in water (180 μM) at 298 K.

**Fig. 7:**
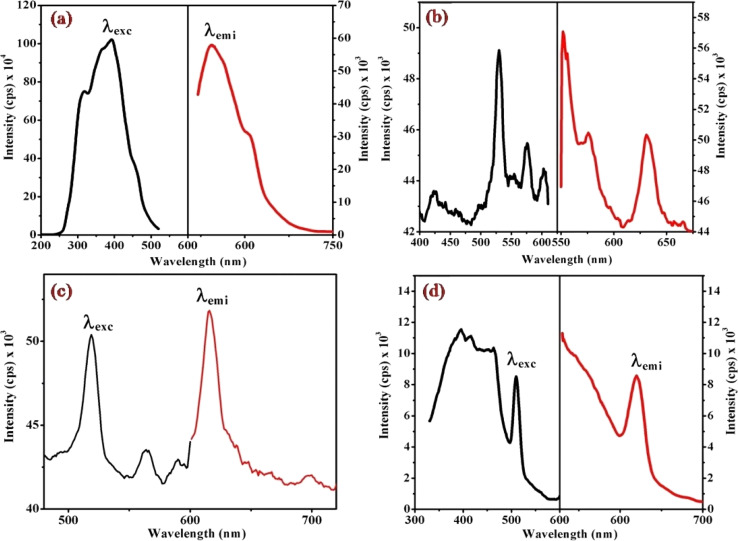
Displays the steady-state excitation and emission spectra of complexes (a) (1), (b) (3), (c) (5), and (d) (6), recorded at *λ*_max_ = 520 nm in water (298 μM) at 298 K.

Fig. 6, 7 and S3† illustrate the characteristic steady-state emission spectra of all prepared complexes (1) to (6) in water (∼180 μM). The emission spectra exhibit maxima at approximately *λ*_emi_ = 341 nm (50.12 × 10^3^ cps), monitored in the wavelength range from 300 to 500 nm, under excitation at *λ*_exc_ = 315 nm (monitored wavelength ranging from 200 to 350 nm). Additionally, emission peaks at around *λ*_emi_ = 620 nm (8.69 × 10^3^ cps) are observed, monitored in the wavelength range from 520 to 700 nm, upon excitation at *λ*_exc_ = 520 and 564 nm (monitored wavelength ranging from 400 to 600 nm). A comprehensive compilation of all emission data is provided in Table 1.

**Table 1:** Photophysical data for complexes (1)–(6) in water (180 μM) at 298 K

Complexes	PL recorded at *λ*_max_ = 256 nm		PL recorded at *λ*_max_ = 520 nm	
	λ_em_ (nm)	Intensity (cps) × 10^3^	λ_em_ (nm)	Intensity (cps) × 10^3^
(1)	396.60	77.99	544.60	58.21
	—	—	607.60	31.08
(2)	339.80	132.25	541.70	70.04
	412.60	95.68	563.80	63.17
	432.40	95.91	618.40	62.08
(3)	341.40	50.12	552.10	57.19
	—	—	576.50	50.59
	—	—	635.70	55.56
(4)	400.30	11.76	619.90	8.69
(5)	538.90	1.73	615.90	51.88
(6)	399.10	11.79	619.90	8.70

When examining the fluorescence behavior of complexes (1) through (6), we observed no precise systematic alterations in emission characteristics. However, a discernible trend emerged wherein there was an approximate lengthening of emission wavelengths from complex (1) with R = 4-MeNH ligand to complex (6) with R = 4-CN ligand in the sixth ligand of RPy within our prepared complexes. This suggests that the presence of a releasing group in RPy results in shorter emission wavelengths compared to a withdrawing group, as detailed in Table 1. While this indicates that the sixth ligand does modestly influence the emission behavior of our prepared complexes, it does not exhibit a consistent pattern of change across the series.

The origin of the emission bands can be attributed to the spin-allowed transition (^1^A_1_ → ^1^T_2g_) state emission, with a secondary weak emission band stemming from the second spin-allowed transition (^1^A_1_ → ^1^T_1g_). Additional insights into the emission decay kinetics were gleaned through single photon counting emission measurements on the cobalt complexes. Intriguingly, the kinetics observed in air-equilibrated water solutions exhibit a triphasic nature, suggesting a multifaceted emission process. These emission studies not only contribute to the understanding of the photophysical properties of the cobalt(iii) complex but also offer valuable data for unraveling the intricate processes underlying its emission behavior in different excitation regimes.

**Fig. 8:**
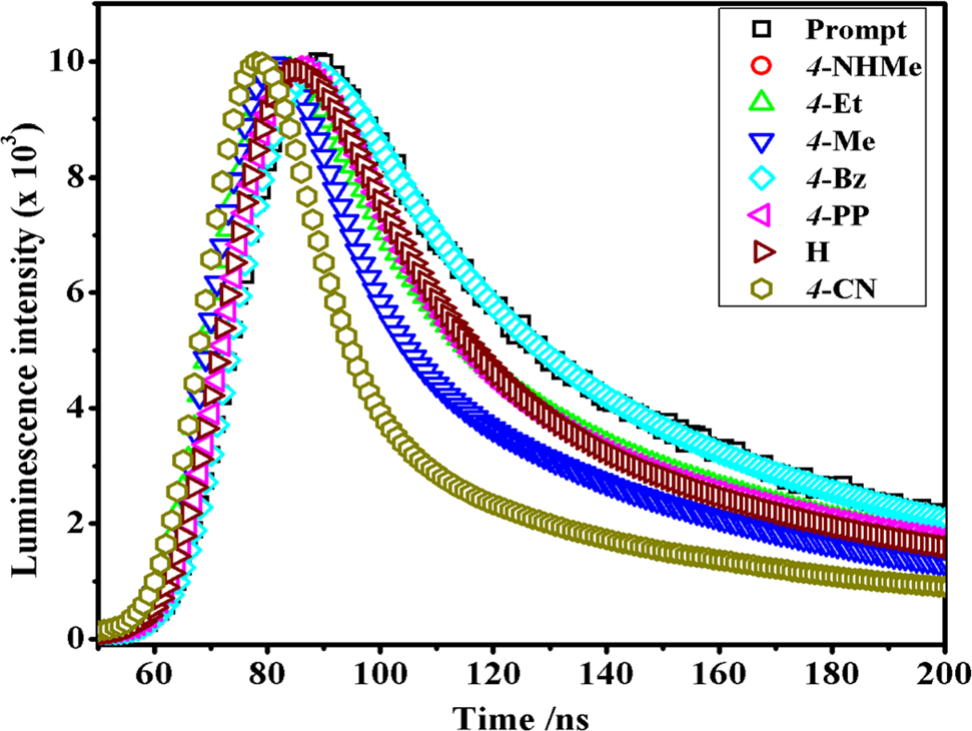
Comparison of time-resolved luminescence profiles for [Co(tn)_2_(Rpy)Br]^2+^ complexes in water solution (∼298 μM) at 298 K.

In the luminescence lifetime experiments, the recorded data exhibit triple exponential decays, as illustrated by the example fit presented in Fig. 8. Notably, the sum of the squares of residuals indicates a random distribution over the regions used to determine lifetimes, suggesting a well-fitted model as the deviations are evenly distributed around zero. The obtained luminescence lifetime results provide insightful information about the excited-state dynamics of the [Co(tn)_2_(Rpy)Br]^2+^ ion. The triple-exponential decays observed in the data suggest the presence of at least three distinct excited-state species. This can be rationalized by considering different spatial locations or conformations of the [Co(tn)_2_(Rpy)Br]^2+^ ion within the studied environment.

Table 2 outlines the luminescence lifetime decay characteristics for the [Co(tn)_2_(Rpy)Br]^2+^ ion. The triple exponential decays indicate the coexistence of multiple excited-state species, shedding light on the complex nature of the luminescent behavior exhibited by the investigated cobalt(iii) complex. This comprehensive analysis of luminescence lifetime data not only contributes to a nuanced understanding of the excited-state dynamics but also underscores the intricate nature of the photochemical processes governing the [Co(tn)_2_(Rpy)Br]^2+^ ion.

**Table 2:** Lifetime data for complexes (1)–(6) in water (180 μM) at 298 K

Complexes	*τ*_1_ (ns)	*τ*_2_ (ns)	*τ*_3_ (ns)	*χ* ^2^
(1)	0.701	4.348	16.172	1.03
(2)	0.767	4.382	16.040	1.05
(3)	3.280	0.359	13.933	1.12
(4)	1.028	4.639	8.596	1.03
(5)	0.917	4.588	1.201	1.11
(6)	0.827	4.448	1.251	1.18

### Magnetic measurement of *cis*-[Co(tn)_2_(Rpy)Br]Br_2_ complexes

**Fig. 9:**
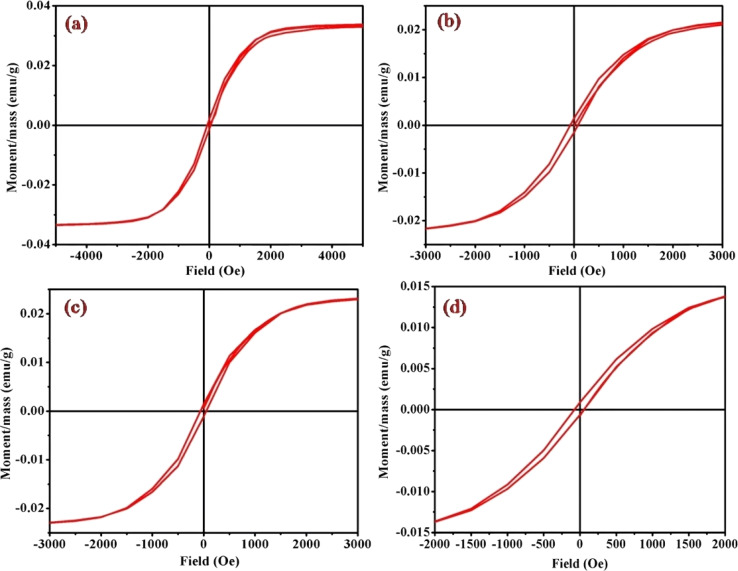
Magnetic hysteresis loops for complexes (a) (1), (b) (3), (c) (5), and (d) (6), measured at 298 K. Note the varied axis scales. All data have been corrected for diamagnetism. The correlation between the molar magnetic susceptibility (*χ*_mol_) and the acidity constant (p*K*_a_) of Rpy provides valuable insights into the magnetic properties of the complexes (1)–(6) in Table 4. The observed linear relationship, expressed as *χ*_mol_ = 2.02 × 10^−4^ – 0.0551 cm^3^ mol^−1^ when p*K*_a_ = 9.70–1.90, indicates that the magnetic susceptibility increases linearly with a decrease in acidity constant. This intriguing correlation suggests a direct influence of the electronic nature of the axial ligand (Rpy) on the magnetic behavior of the complexes.

Magnetic hysteresis loops, representing a distinctive fingerprint of ferromagnetism, were experimentally observed as a crucial aspect of our investigation. The vibrating sample magnetometry (VSM) technique was employed to characterize the hysteresis curves of complexes (1)–(6) in a DC magnetic field. Fig. 9 and S4† illustrates the hysteresis loops for the cobalt(iii) complexes, plotting moment/mass (emu g^−1^) against field (Oe). The presence of hysteresis loops in the magnetic measurements strongly suggests the manifestation of ferromagnetic behavior within the cobalt(iii) complexes. Intriguingly, the intrinsic coercivity, remanent magnetization, and molar magnetic susceptibility values, derived from the VSM analysis and detailed in Table 3, reinforce this observation. The results collectively point towards the weak ferromagnetic nature of all [Co(tn)_2_(Rpy)Br]Br_2_ complexes, coupled with significant magnetization.

**Table 3:** Magnetic characteristics of complexes (1)–(6) obtained from vibrating sample magnetometer

Complexes	p*K*_a_ (Rpy)	*M*_s_ × 10^−3^ (emu g^−1^)	*χ*_mass_ × 10^−3^ (cm^3^)	*χ*_mol_ × 10^−4^ (cm^3^)	Hc, Oe	*M*_r_ ×10^−3^ (emu g^−1^)	*μ*_r_ (emu g^−1^)
(1)	9.70	33.82	2.09	2.02	55	1.65	1.002
(2)	6.02	15.59	3.41	3.38	82	1.18	1.003
(3)	6.02	23.19	4.10	3.96	78	1.50	1.004
(4)	5.59	13.20	32.80	36.24	65	0.91	1.033
(5)	5.25	23.94	37.00	34.85	57	1.28	1.024
(6)	1.90	16.06	558.00	551.50	65	0.76	1.016

A closer examination of the magnetic data unveils a consistent variation in molar magnetic susceptibility, intricately linked to the unique nature of the complex ion [Co(tn)_2_(Rpy)Br]Br_2_. Specifically, this variation correlates with the presence of the sixth ligand in the coordination environment, denoted as R in the Rpy ligand. The nature of R, influenced by the attachment of electron-withdrawing or donating groups, plays a crucial role in modulating the magnetic properties of the complexes. Remarkably, despite R being distantly positioned in relation to the metal center, the magnetic behavior of the complexes exhibits a discernible relationship with the acidity constant (p*K*_a_). This intriguing correlation further emphasizes the intricate interplay between the molecular structure and the magnetic properties observed in complexes (1)–(6).

Examining the intrinsic coercivity (Hc) in the range of 55 to 82 Oe provides further evidence of the magnetic behavior within the complexes (1)–(6). The room temperature magnetic hysteresis loop measurements confirm the ferromagnetic nature of the cobalt(iii) complexes, with variations in magnetic character correlating with the basicity of the axial ligand. This observation aligns with similar findings reported for certain low-spin cobalt(iii) complexes.^40^

Interestingly, the anticipated non-linear relationship between the magnetic properties and the substituents in the pyridyl ligand is revealed to be more complex than initially expected. Comparable investigations have demonstrated correlations between the demagnetization properties of mixed-ligand nicotinate *N*-oxide metal(ii) complexes, emphasizing the intricate nature of such relationships.^40^ Furthermore, existing literature highlights clear correlations between d–d band positions and the electronic effects of ligand substituents. Reports on metal complexes with pyridyl amine also reinforce the association of physicochemical properties with the electronic nature of substituents.^41^

The magnetic characteristics of the complexes (1)–(6) exhibit a nuanced interplay with the electronic properties of the axial ligand and its basicity. The non-linear correlation between molar magnetic susceptibility and p*K*_a_ values adds a quantitative dimension to the understanding of these relationships. The observed ferromagnetic behavior and its variation underscore the complex nature of the magnetic properties in coordination complexes, providing valuable insights for further investigations in this intriguing field of study.

### Cobalt(iii)-R-pyridyl complexes: photoexcitation on nanoscale TiO_2_

Upon UV-light excitation (*λ* = 254 nm), the nano-TiO_2_//cobalt(iii)-(Rpy) surface compound initiated IET reactions. The efficiency of IET was observed to be dependent on the coordination environment of the complex, with a notable influence from the nature of the Rpy ligand. Building upon our previous investigation of Co(iii)-alkyl amine complexes with TiO_2_, where we proposed a mechanism for interfacial electron transfer involving the formation of Co^2+^ ions implanted in nanocrystalline TiO_2_, the same mechanism is applied in this study.^42^ Our investigation encompasses the characterization of electronic excitations, electron injection time scales, and interfacial electron transfer mechanisms, along with the photoefficiency of IET and the photo-degradation of 2-propanol. Specifically, we concentrate on TiO_2_ surfaces modified by Co(iii)-pyridyl complexes tethered *via* nitrogen linkers. The focal point lies in detailing the electronic excitations and injection time scales, which are influenced by the nature of the molecular adsorbates and the modes of attachment.

**Fig. 10:**
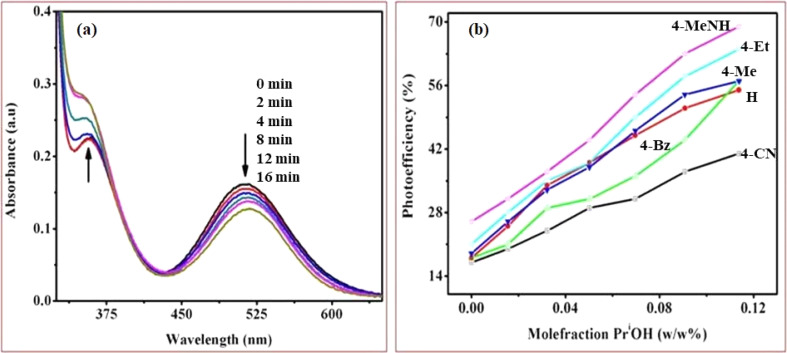
(a) UV-vis absorption spectra were obtained during the photocatalytic irradiation of the [Co(tn)_2_(4-Etpy)Br]^2+^ complex ion with nano-TiO_2_ suspension in pure water at different time points and (b) Graph illustrating the correlation between the mole fraction of water (w/w % PriOH + water) and photoefficiency, determined through spectrophotometer measurements during the photo-irradiation of a series of [Co(tn)_2_(Rpy)Br]^2+^ complex ions (R = 4-CN, H, 4-Bz, 4-Me, 4-Et, and 4-MeNH). The complex concentration was maintained at 1.68 × 10^−3^ M in water/2-propanol mixtures with nanocrystalline TiO_2_. Experimental conditions included an ionic strength of 1 M NaNO_3_, pH ∼7, and a temperature of 298 K.

The cobalt(iii)-Rpy complex serves as a proficient UV light absorber, yet it undergoes decomposition upon prolonged light exposure, primarily attributed to ligand-to-metal charge transfer (LMCT) bands at 356 nm. Introduction of nano-TiO_2_ (anatase) significantly enhances the complex degradation efficiency, resulting in a higher generation of cobalt(ii). In Fig. 10(a), UV-vis absorption spectra for [Co(tn)_2_(4-Etpy)Br]^2+^ complex ion is presented at distinct irradiation time intervals. Notably, a blue shift in the absorption maxima occurs at *λ* = 357 → 351 nm, accompanied by a red shift at *λ* = 512 → 518 nm.

The absence of an isosbestic point in the UV-vis spectra indicates complex decomposition during the reduction process, suggesting perturbation of the Co(iii) center due to IET. The photolytic solutions exhibit a photoefficiency of Co^2+^ formation by TiO_2_ (e_cb_^−^/h_vb_^+^)/scavenger of [Co(tn)_2_(4-Etpy)Br]^2+^, which increases with the concentration of 2-propanol, showing heightened activity at higher concentrations (Fig. 10(b)). This suggests that the observed photoefficiency (as detailed in Table 5) of Co^2+^ formation is a summation of individual electron transfer reactions, specifically involving (i) excited nano-TiO_2_:[Co(tn)_2_(Rpy)Br]^2+^ + nano-TiO_2_ + *hν*(*λ* = 254 nm) → Co^2+^ + products and (ii) ligand-to-metal charge transfer transition in the cobalt(iii) complex: [Co(tn)_2_(Rpy)Br]^2+^ + *hν*(*λ* = 254 nm) → Co^2+^ + products. However, the former path is predominant and necessitates further in-depth investigation.

The nanocrystalline titania has a more pronounced impact on the photoreduction of the [Co(tn)_2_(Rpy)Br]^2+^ complex compared to its polycrystalline counterpart in Table 4. Nanocrystalline materials, characterized by a grain size typically less than 100 nm, exhibit unique properties due to the significant volume of their microstructure composed of interfaces, particularly grain boundaries. In contrast to conventional coarse-grained polycrystalline materials, nanocrystalline counterparts often demonstrate enhanced and distinct properties.^43^ In polycrystalline wide band gap oxide semiconductors like ZnO and TiO_2_, the photoconductivity (PC) decay is observed to be notably slow, persisting over extended periods ranging from hours to days.^42,44^ This phenomenon, often termed ‘persistent PC,’ has prompted various proposed explanation.^45–47^ In some instances, models have been employed to fit experimental data, aiding in the determination of electronic parameters such as the energy distribution of charge carrier traps. Previous studies on photocatalytic degradation of pollutants have explored different approaches. For instance, investigations using Co(ii)-tetrasulfophthalocyanine grafted on TiO_2_*via* a silane reagent have been documented, along with studies involving polycrystalline TiO_2_ samples impregnated with Cu(ii)-phthalocyanine.^48,49^ These findings underscore the importance of nanocrystalline materials in influencing photoreduction processes and the distinct behavior exhibited by such materials, shedding light on potential advancements in photocatalytic applications.

**Table 4:** Photoefficiency PE (%) of formation of Co(ii) upon *λ* = 254 nm irradiation of complexes (1)–(6) in aqueous 2-propanol at 298 K. Complex concentration = 1.68 × 10^−3^ M, ionic strength 1 M NaNO_3_^a^

p*K*_a_ value of Rpy	Complexes	Catalyst used	PE (%) in water/PriOH (w/w)%						
			100/0	95/5	90/10	85/15	80/20	75/25	70/30
1.90 (R = 4-CN)	(6)	—	14 ± 0.5	18 ± 1.0	19 ± 1.0	21 ± 0.7	26 ± 0.8	28 ± 0.9	32 ± 0.6
		Poly-TiO_2_	16 ± 1.0	17 ± 0.5	21 ± 1.0	24 ± 0.8	29 ± 0.7	31 ± 0.6	37 ± 0.5
		Nano-TiO_2_	17 ± 0.7	20 ± 0.9	24 ± 0.5	29 ± 0.8	31 ± 0.6	37 ± 1.0	41 ± 1.4
5.25 (R = H)	(5)	—	15 ± 0.8	21 ± 0.7	29 ± 1.0	34 ± 0.5	38 ± 1.4	40 ± 0.9	42 ± 1.2
		Poly-TiO_2_	14 ± 0.6	24 ± 1.0	32 ± 0.8	35 ± 0.8	41 ± 0.7	44 ± 0.9	48 ± 1.2
		Nano-TiO_2_	18 ± 1.0	25 ± 0.7	34 ± 0.6	39 ± 1.0	45 ± 0.5	51 ± 0.9	55 ± 0.5
5.59 (R = 4-Bz)	(4)	—	12 ± 0.8	19 ± 1.0	24 ± 0.6	28 ± 0.7	32 ± 0.8	38 ± 0.5	41 ± 1.2
		Poly-TiO_2_	16 ± 0.6	23 ± 0.8	28 ± 1.0	30 ± 1.4	34 ± 0.5	38 ± 0.9	49 ± 0.8
		Nano-TiO_2_	18 ± 0.6	21 ± 0.8	29 ± 0.7	31 ± 0.5	36 ± 1.0	44 ± 0.9	57 ± 1.4
6.02 (R = 4-Me)	(3)	—	12 ± 1.0	19 ± 1.0	26 ± 0.5	29 ± 0.8	34 ± 0.9	38 ± 0.8	49 ± 1.4
		Poly-TiO_2_	17 ± 1.0	23 ± 0.5	29 ± 0.6	34 ± 0.8	37 ± 0.9	45 ± 0.9	52 ± 0.7
		Nano-TiO_2_	19 ± 0.7	26 ± 0.5	33 ± 1.0	38 ± 0.6	46 ± 0.8	54 ± 1.4	57 ± 0.9
6.02 (R = 4-Et)	(2)	—	18 ± 1.2	24 ± 0.6	28 ± 1.0	31 ± 0.5	36 ± 1.4	41 ± 0.8	49 ± 0.5
		Poly-TiO_2_	19 ± 1.0	27 ± 1.0	32 ± 0.9	37 ± 0.8	45 ± 0.5	48 ± 0.8	53 ± 1.4
		Nano-TiO_2_	21 ± 0.6	28 ± 0.5	35 ± 1.0	39 ± 0.9	49 ± 0.8	58 ± 0.5	64 ± 0.7
9.70 (R = 4-MeNH)	(1)	—	19 ± 0.8	24 ± 0.5	29 ± 0.5	32 ± 1.0	46 ± 1.0	52 ± 0.8	53 ± 0.9
		Poly-TiO_2_	23 ± 0.7	28 ± 0.8	32 ± 0.5	38 ± 0.6	49 ± 1.0	58 ± 0.9	61 ± 0.5
		Nano-TiO_2_	26 ± 0.8	31 ± 0.6	37 ± 0.7	44 ± 0.5	54 ± 1.0	63 ± 0.9	69 ± 0.8

a Photo-oxidation of 2-propanol into acetone.

**Fig. 11:**
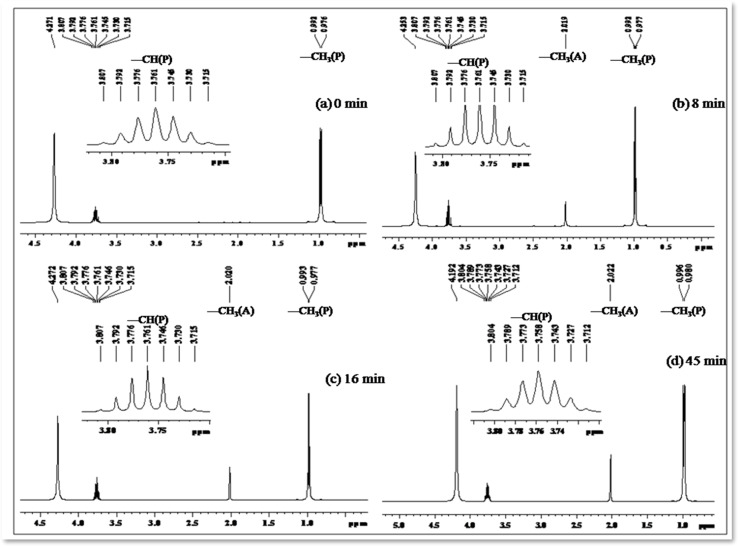
^1^H NMR spectrum (in DMSO-d_6_) depicting the decomposition profile of 2-propanol in the presence of nano-TiO_2_/[Co(tn)_2_(4-Etpy)Br]^2+^/UV-light (254 nm) at different time intervals: (a) 0, (b) 8, (c) 16, and (d) 45 minutes, respectively. ‘A’ and ‘P’ represent acetone and 2-propanol, respectively. The inset highlights the –CH signal of 2-propanol in the region 3.7–3.83 ppm.

Photo-oxidation of 2-propanol leading to acetone production has been extensively studied. Previous literature reports highlight the remarkable catalytic activities of cobalt-doped TiO_2_ in the degradation of acetaldehyde, 2-chlorophenol, and 2,4-dichlorophenol in aqueous solutions.^50–53^ In our investigation, we systematically monitored the photo-oxidation process using NMR measurements, revealing a progressive increase in the signal intensity corresponding to the growth of the acetone peak as a function of light dosage.^54^Table 5 presents ^1^H NMR signals associated with 2-propanol, identified at *δ* = 1.0 to 1.1 ppm (–CH_3_) and *δ* = 3.8 to 3.93 ppm (–CH), both before the initiation of photo-oxidation and at specific intervals during irradiation periods (refer to Fig. 11). Notably, a distinct ^1^H NMR signal at *δ* = 2.04 ppm emerges, signifying the formation of acetone, and its intensity (refer to Table 5) considerably increases over extended irradiation times.^55^

**Table 5:** ^1^H NMR data illustrating the production of acetone upon irradiation at *λ* = 254 nm of [Co(tn)_2_(4-Etpy)Br]^2+^ at 298 K. ‘A’ and ‘P’ represent acetone and 2-propanol, respectively. DMSO-d_6_ was used as the NMR solvent^a^

Irradiating time (min)	2× –CH_3_(d) P *δ* (ppm)		–CH(m) P *δ* (ppm)							2× –CH_3_(s) A *δ* (ppm)
0	1.014	1.002	3.723	3.738	3.753	3.769	3.784	3.799	3.814	—
8	1.001	1.017	3.739	3.755	3.770	3.785	3.801	3.816	3.831	2.050
16	1.006	1.022	3.717	3.732	3.747	3.763	3.778	3.793	3.808	2.040
45	1.007	1.023	3.724	3.740	3.755	3.770	3.786	3.801	3.816	2.045

a Mechanism of photoinduced electron transfer reaction.

The integrated intensity of the acetone signals demonstrates a steady growth, accompanied by a gradual decrease in the signals corresponding to –CH and –CH_3_ (2-propanol). This observation implies that acetone originates from the oxidation of 2-propanol, a process catalyzed by the scavenging of valence band holes by alcohol, expressed as Co–TiO_2_(h^+^) + (CH_3_)_2_CHOH → CH_3_COCH_3_. These findings underscore the dynamic nature of the photo-oxidation process and provide valuable insights into the mechanistic aspects of acetone generation from 2-propanol oxidation.

Tables 4 and 5 clearly reveal that excited nanocrystalline TiO_2_ exhibits superior catalytic activity, a phenomenon intricately linked to the surface–substrate interaction. The surface affinity of molecules/ions presents a competitive aspect, necessitating the inevitable formation of a polycrystalline (or) nanocrystalline TiO_2_//cobalt(iii)-(Rpy) surface compound. The augmentation in photocatalytic reduction stems from two primary factors: (i) Rpy's ability to modify the surface affinity of [Co(tn)_2_(Rpy)Br]^2+^ ions with the nano-TiO_2_ surface, and (ii) photoexcitation inducing the formation of microdomains in nano-TiO_2_ with distinctive hydrophobic/hydrophilic behavior.^56,57^

The incorporation of the sixth ligand (Rpy) in [Co(tn)_2_(Rpy)Br]^2+^, characterized by a hydrophobic tail, introduces variations in the surface adherence of the complex ion to the nano-TiO_2_ surface. However, it is important to note that the adsorption process is constrained by thermodynamic considerations. To provide a comprehensive understanding of these observations, we propose the following mechanistic equations (eqn (1) and (3)):

1 nano-TiO_2_ + [Co(tn)_2_(Rpy)Br]^2+^ → nano-TiO_2_//[Co(tn)_2_(Rpy)Br]^2 +^

2 nano-TiO_2_(h^+^,VB)/(h^+^,tr) + nano-TiO_2_(e^−^,CB)/(e^−^,tr) + (Co^3+^)+(CH_3_)_2_CHOH → CH_3_COCH_3_ + Co_surf_^2+^ + Co^2+^

3 nano-TiO_2_(h^+^,VB)/(h^+^,tr) + nano-TiO_2_(e^−^,CB)/(e^−^,tr) + (Co^3+^) + (CH_3_)_2_CHOH → CH_3_COCH_3_ + Co_surf_^2+^ + Co_aq._^2+^

These equations elucidate the intricate processes involved in the interaction between nanocrystalline TiO_2_ and [Co(tn)_2_(Rpy)Br]^2+^ complex ion, and the subsequent photocatalytic reduction leading to the production of acetone and the involvement of surface-bound Co^II^ species.

### p*K*_a_ dependent photoreduction of Co(iii)-Rpy complexes

**Fig. 12:**
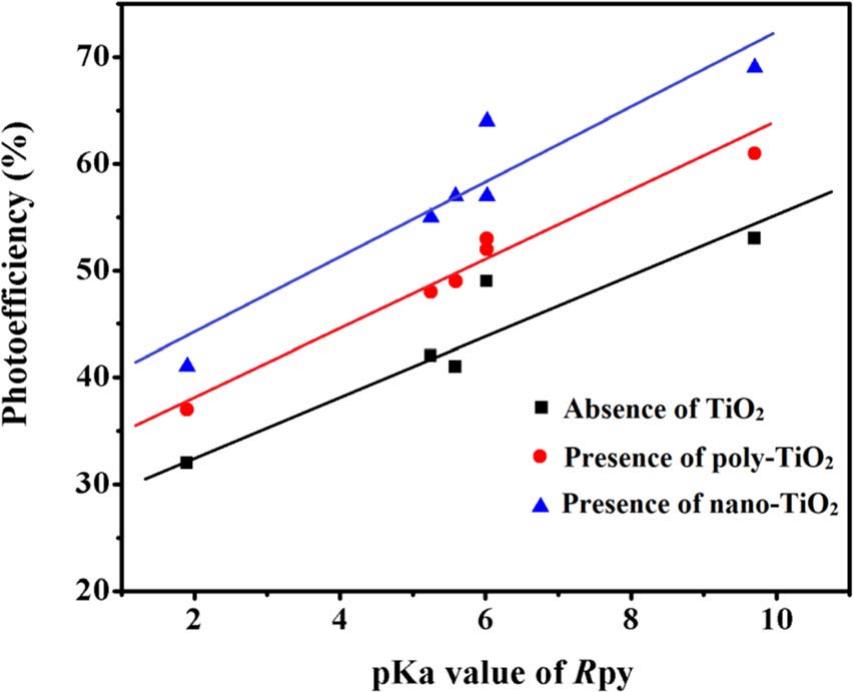
Linear relationship between photoefficiency and p*K*_a_ value of aryl amine (R) in [Co(tn)_2_(Rpy)Br]^2+^ under varied conditions: absence of TiO_2_, presence of poly-TiO_2_, and nano-TiO_2_ in 70/30 (w/w)% water/PriOH.

The photoreduction of Co(iii)-Rpy complexes exhibits a notable p*K*_a_ dependency, as evidenced in this investigation. A linear relationship between the photoefficiency (%) of [Co(tn)_2_(Rpy)Br]^2+^/TiO_2_ suspension and the acidity constant (p*K*_a_) of the Rpy ligand is observed, as illustrated in Fig. 12. The linear regression analysis of photoefficiency (%) against p*K*_a_ reveals a slope that signifies the influence of the electron-donating or electron-withdrawing nature of the Rpy ligand on the photoefficiency. Specifically, the electron-donating group in Rpy, characterized by a higher p*K*_a_ (>5.25), is found to enhance photoefficiency (%). Conversely, the electron-withdrawing group in Rpy, with a lower p*K*_a_ value (p*K*_a_ = 1.90), diminishes the photocatalytic behavior. This empirical observation underscores the significant impact of the coordination environment of the transition metal ion's metal center on the photochemical characteristics of the complex.

In addition to the p*K*_a_-dependent trends, it is noteworthy to explore the mechanistic underpinnings of how the electron-donating or withdrawing nature of Rpy influences the photoefficiency, providing a comprehensive understanding of the observed phenomena. Further investigations into the molecular–level interactions and electronic properties could contribute valuable insights to the design and optimization of photoactive complexes for enhanced catalytic performance.

The photogeneration efficiency (PE %) of Co^2+^ in our study exhibits intriguing dynamics, displaying a dual dependence: (i) on the 2-propanol content and (ii) a correlation with variations in the Rpy ligand of the complex. This non-consistency is attributed to the intricate interplay between nanoparticle surface characteristics and cobalt(iii)-Rpy affinity, influencing subsequent interfacial electron transfer processes. The accumulation of the cobalt(iii) complex onto the surface of nano-TiO_2_, forming a compact layer, is significantly influenced by the blocking effect of Rpy, dictated by its electron-withdrawing and electron-donating properties. Notably, Pellizzetti and colleagues have previously demonstrated a substantial alteration in the distribution of aromatic intermediates on the surface of TiO_2_, underscoring the impact of surface interactions on catalytic processes.^58^

In Fig. 12, the relationship between photoefficiency and the p*K*_a_ value of the aryl amine of the Rpy ligand for six [Co(tn)_2_(Rpy)Br]^2+^ complexes is elucidated. Within this limited set of complexes, a discernible correlation emerges between PE and the p*K*_a_ value of the ligand. Interestingly, there is a noticeable enhancement in PE with the aromatic ring's electronic nature of the Rpy ligand. This enhancement seems to approach a limiting value asymptotically, suggesting a saturation point in the influence of the aromatic nature on the photogeneration efficiency of Co^2+^. This intricate interplay between ligand properties, surface interactions, and electron transfer processes underscores the complexity of the system. Further exploration of a broader range of complexes and in-depth mechanistic investigations could unveil additional nuances, paving the way for fine-tuning and optimizing the photoreduction processes for enhanced catalytic performance.

## Conclusion

This investigation presents a novel mechanochemical synthesis of mixed ligand complexes, specifically the *cis*-[Co(tn)_2_(Rpy)Br]Br_2_ type. The comprehensive characterization, involving spectral measurements and single crystal X-ray diffraction analysis, confirmed the structural integrity of these complexes. Intriguingly, the emission and magnetic properties exhibited a ferromagnetic character influenced by the choice of the sixth ligand in the Co(iii)-complex environment. Exploring the electron transfer processes, the study demonstrated efficient electron transfer to the cobalt(iii) center utilizing TiO_2_ nanoparticles under UV-light irradiation. The adsorption characteristics of *cis*-[Co(tn)_2_(Rpy)Br]Br_2_ in aqueous 2-propanol led to the formation of surface compounds. UV excitation of the nano-TiO_2_//cobalt(iii)-(Rpy) surface compound initiated an IET reaction, dependent on the coordination environment of the complex, particularly the Rpy ligand. Spectral analysis revealed the photoefficiency of Co_aq._^2+^ formation, with UV irradiation of the anatase surface demonstrating potent adsorption capabilities, facilitating efficient electron transfer to the Co(iii) center and resulting in high photoefficiency of Co(ii) formation. A proposed model for electron transfer considered the overlap of the TiO_2_ conduction band with the acceptor level (Co center) and the electronic coupling of the donor level (localized on Ti center) with the acceptor level (Co center). These pathways imply the accumulation of electrons, available for the reduction of the adhered complex ion. This study not only sheds light on the role of the Rpy moiety in modifying the TiO_2_-cobalt(iii)-Rpy compound's compact structure and the redox power of the semiconductor surface but also proposes a mechanism for interfacial electron transfer reactions. The IET process was verified by the conversion of 2-propanol into acetone, as verified by ^1^H NMR technique. These findings contribute novel insights to the field, emphasizing the intricate interplay of ligand effects in coordination chemistry and the potential for advancing photocatalytic applications.

## Conflicts of interest

There are no conflicts to declare.

## Supplementary Material

RA-014-D4RA02648A-s001
