# Supplementary material for: Unveiling photoinduced electron transfer in cobalt(iii)-R-pyridine complexes anchored to anatase nanocrystals: photoluminescence and magnetic studies
Source: RSC Adv. 2024 May 28;14(24):17218–33. doi: 10.1039/d4ra02648a (PMC11131157; doi:10.1039/d4ra02648a)
Supplement: RA-014-D4RA02648A-s001 [file RA-014-D4RA02648A-s001.pdf]

Electronic Supplementary Material (ESI) for RSC Advances.
This journal is © The Royal Society of Chemistry 2024

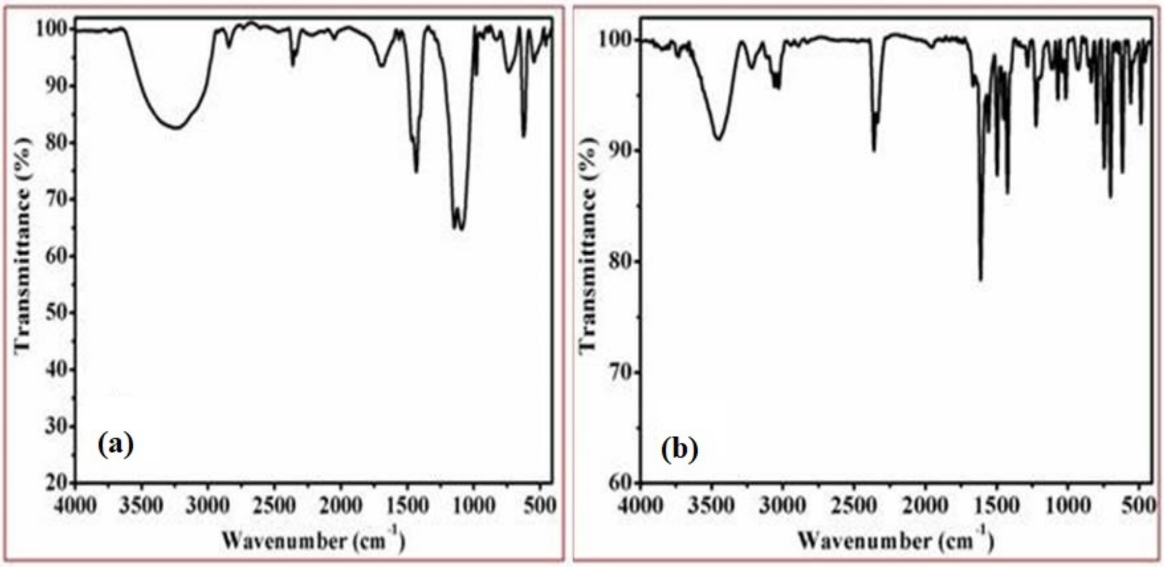

Figure S1 FTIR spectra of (a) complex **(2)** and (b) complex **(4)** measured with KBr at 298 K. where R = (a) 4-Et, (b) 4-Bz.

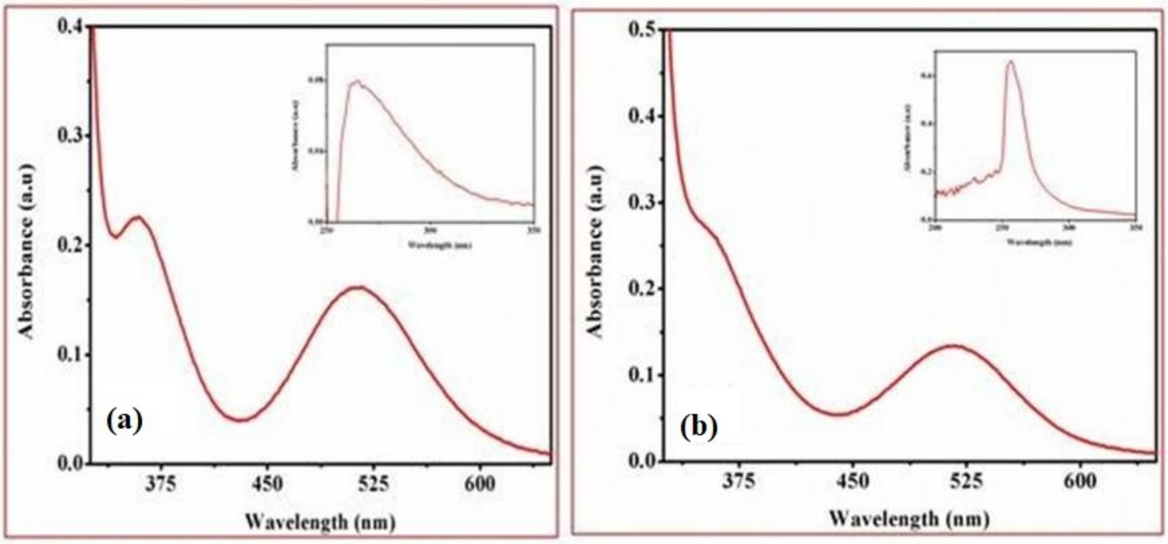

Figure S2 UV-vis spectra of (a) complex **(2)** and (b) complex **(4)** measured in water at 298 K.

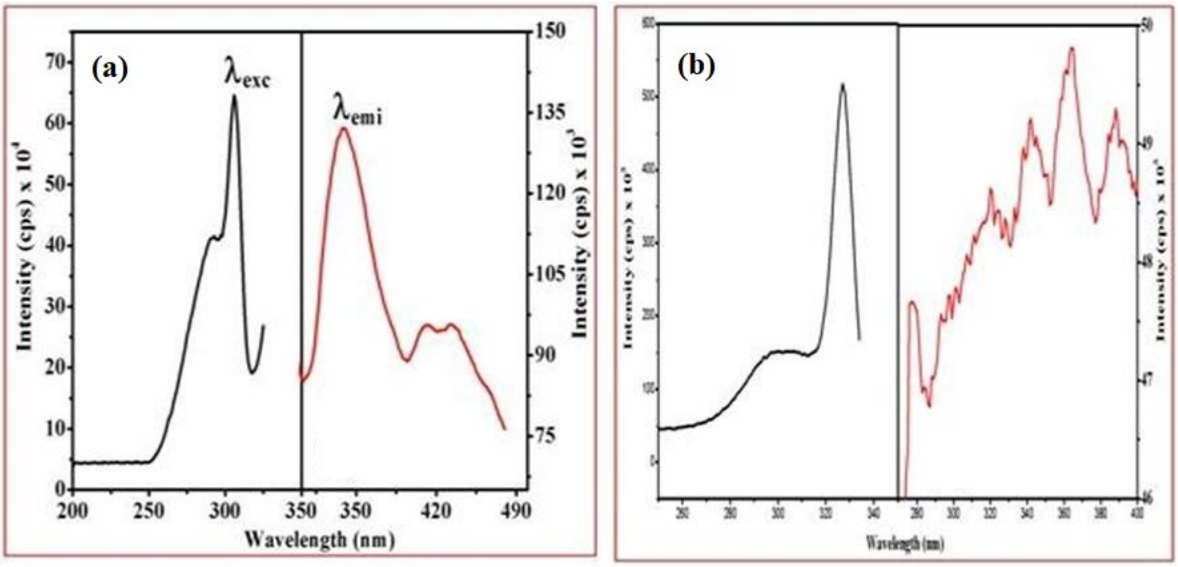

Figure S3 Steady state excitation and emission spectra of (a) complex **(2)** and (b) complex **(4)** measured at λ_{max} = 256 nm in water (~2 μM) at 298 K.

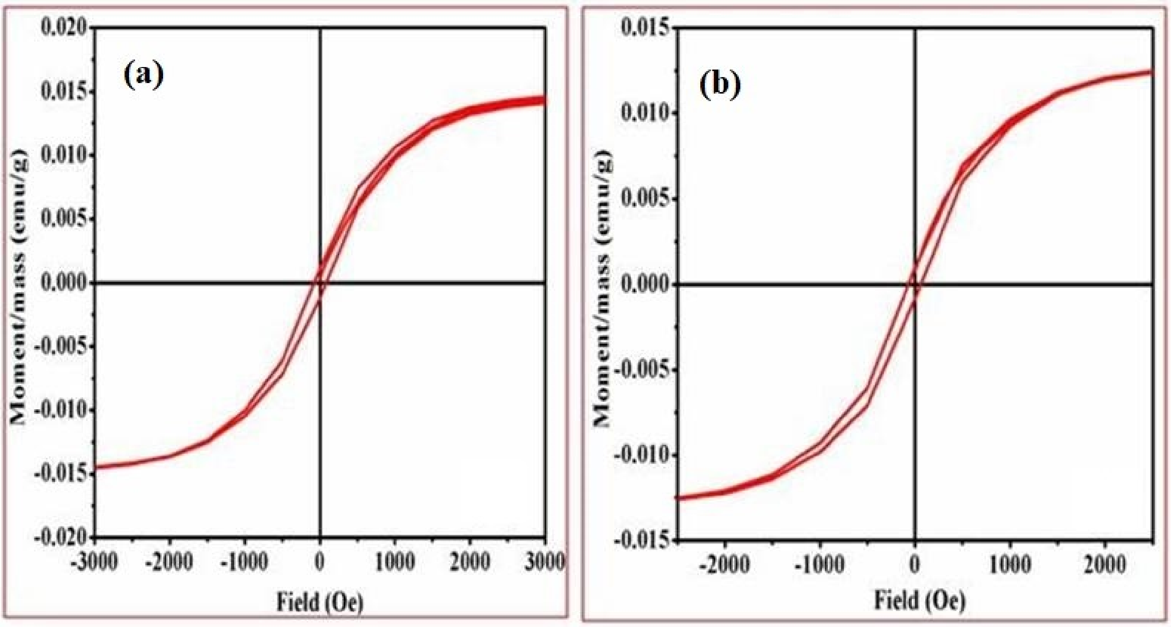

Figure S4 Magnetic hysteresis loop of (a) complex **(2)** and (b) complex **(4)** measured at 298 K.
